# Some new sharp bounds for the spectral radius of a nonnegative matrix and its application

**DOI:** 10.1186/s13660-017-1536-3

**Published:** 2017-10-19

**Authors:** Jun He, Yan-Min Liu, Jun-Kang Tian, Xiang-Hu Liu

**Affiliations:** School of Mathematics, Zunyi Normal College, Zunyi, Guizhou 563006 P.R. China

**Keywords:** 05C50, 05C35, 05C20, 15A18, nonnegative matrix, graph, digraph, spectral radius

## Abstract

In this paper, we give some new sharp upper and lower bounds for the spectral radius of a nonnegative irreducible matrix. Using these bounds, we obtain some new and improved bounds for the signless Laplacian spectral radius of a graph or a digraph.

## Introduction

Let $G=(V,E)$ be a graph with vertex set $V(G)=\{v_{1}, \ldots, v_{n}\}$ and edge set $E(G)$. Let $N=\{1, \ldots, n\}$, for $i \in N$. We assume that $d_{i}$ is the degree of vertex $v_{i}$. Let $D(G) = \operatorname{diag}(d_{1}, d_{2}, \ldots, d_{n})$ be the degree diagonal matrix of the graph *G* and $A(G) = (a_{ij})$ be the adjacency matrix of the graph *G*. Then the matrix $Q(G) = D(G)+ A(G)$ is called the signless Laplacian matrix of the graph *G*. The largest modulus of eigenvalues of $Q(G)$ is denoted by $\rho(G)$, which is also called the signless Laplacian spectral radius of *G*.

Let $\overrightarrow{G}=(V,E)$ be a digraph with vertex set $V(\overrightarrow{G})=\{v_{1}, \ldots, v_{n}\}$ and arc set $E(\overrightarrow{G})$. Let $d_{i}^{+}$ be the out-degree of vertex $v_{i}$, $D(\overrightarrow{G}) = \operatorname{diag}(d_{1}^{+}, d_{2}^{+}, \ldots, d_{n}^{+})$ be the out-degree diagonal matrix of the digraph $\overrightarrow{G}$, and $A(\overrightarrow{G}) = (a_{ij})$ be the adjacency matrix of the digraph $\overrightarrow{G}$. Then the matrix $Q(\overrightarrow{G}) = D(\overrightarrow{G})+ A(\overrightarrow{G})$ is called the signless Laplacian matrix of the digraph $\overrightarrow{G}$. The largest modulus of eigenvalues of $Q(\overrightarrow{G})$ is denoted by $\rho (\overrightarrow{G})$, which is also called the signless Laplacian spectral radius of $\overrightarrow{G}$.

In recent decades, there are many bounds on the signless Laplacian spectral radius of a graph (digraph) [1–3]. Let $m_{i} = \frac{{\sum_{i\sim j} {d_{j} } }}{{d_{i} }}$ be the average degree of the neighbours of $v_{i}$ in *G* and $m_{i}^{+} = \frac{{\sum_{i\sim j} {d_{j}^{+} } }}{{d_{i}^{+} }}$ be the average out-degree of the out-neighbours of $v_{i}$ in $\overrightarrow{G}$. In this paper, we assume that the graph (digraph) is simple and connected (strong connected).

In 2013, Maden, Das, and Cevik [4] obtained the following bounds for the signless Laplacian spectral radius of a graph:

1 $$ \rho(G) \leq\max_{i\sim j} \biggl\{ \frac{d_{i}+ 2d_{j} -1+ \sqrt {(d_{i}-2d_{j}+1)^{2}+ 4d_{i}}}{2} \biggr\} . $$

In 2016, Xi and Wang [5] obtained the following bounds for the signless Laplacian spectral radius of a digraph:

2 $$ \rho(\overrightarrow{G}) \leq\max_{i\sim j} \biggl\{ \frac{d_{i}^{+}+ 2d_{j}^{+} -1+ \sqrt{(d_{i}^{+}-2d_{j}^{+}+1)^{2}+ 4d_{i}^{+}}}{2} \biggr\} . $$

In this paper, we improve the bounds for the signless Laplacian spectral radius of a graph (digraph) that are given in (1) and (2).

## Main result

In this section, some upper and lower bounds for the spectral radius of a nonnegative irreducible matrix are given. We need the following lemma.

### Lemma 2.1

([6])

*Let* *A* *be a nonnegative matrix with the spectral radius* $\rho(A)$ *and the row sum* $r_{1}, r_{2}, \ldots, r_{n}$. *Then* $\mathop{\min} _{1 \le i \le n} r_{i} \le\rho(A) \le\mathop{\max} _{1 \le i \le n} r_{i}$. *Moreover*, *if the matrix* *A* *is irreducible*, *then the equalities hold if and only if*

$$r_{1}=r_{2}= \cdots=r_{n}. $$

### Theorem 2.1

*Let* $A=(a_{ij})$ *be an irreducible and nonnegative matrix with* $a_{ii} = 0$ *for all* $i \in N$ *and the row sum* $r_{1}, r_{2}, \ldots, r_{n}$. *Let* $B = A + M$, *where* $M = \operatorname{diag}(t_{1}, t_{2}, \ldots, t_{n})$ *with* $t_{i} \geq0$ *for any* $i \in N$, $s_{i} = \sum_{j = 1}^{n} {a_{ij} r_{j} }$, $s_{ij} = s_{i}-a_{ij}r_{j}$. *Let* $\rho(B)$ *be the spectral radius of* *B* *and let*

$$f(i,j) = \frac{t_{i}+t_{j}+\frac{s_{ij}}{r_{i}}+\sqrt{ (t_{i}-t_{j}+\frac {s_{ij}}{r_{i}} )^{2}+\frac{4s_{j}a_{ij}}{r_{i}}}}{2}, $$

*for any* $i,j \in N$. *Then*

3 $$ \mathop{\min} _{1 \le i \le n} \mathop{\max_{1 \le j \le n }}_{ j \ne i } \bigl\{ f(i,j),a_{ij}\neq0\bigr\} \le\rho(B) \le\mathop{\max} _{1 \le i \le n} \mathop{\min _{1 \le j \le n }}_{ j \ne i } \bigl\{ f(i,j),a_{ij} \neq0\bigr\} . $$

*Moreover*, *either of the equalities in* (3) *holds if and only if* $t_{i}+\frac{s_{i}}{r_{i}}= t_{j}+\frac{s_{j}}{r_{j}}$ *for any distinct* $i,j \in N$.

### Proof

Let $R = \operatorname{diag}(r_{1}, r_{2}, \ldots, r_{n})$. Since the matrix *A* is nonnegative irreducible, the matrix $R^{-1}BR$ is also nonnegative and irreducible. By the famous Perron-Frobenius theorem [6], there is a positive eigenvector $x =(x_{1}, x_{2}, \ldots, x_{n})^{T}$ corresponding to the spectral radius of $R^{-1}BR$.

Upper bounds: Let $x_{p}>0$ be an arbitrary component of *x*, $x_{q}=\max\{ x_{k}, 1\leq k \leq n\}$. Obviously, $p\neq q$, $a_{pq}\neq0$. By $R^{-1}BRx = \rho(B)x$, we have

4 $$ \rho(B)x_{p}=t_{p}x_{p}+ \sum _{k = 1,k \ne p}^{n} {\frac{{a_{pk} r_{k} x_{k} }}{{r_{p} }}}\leq t_{p}x_{p} + \frac{x_{q}}{r_{p}}\sum _{k = 1}^{n} a_{pk} r_{k}\leq t_{p}x_{p} + \frac{x_{q}s_{p}}{r_{p}}. $$

Similarly, we have

5 $$ \rho(B)x_{q}=t_{q}x_{q}+ \sum _{k = 1,k \ne q}^{n} {\frac{{a_{qk} r_{k} x_{k} }}{{r_{q} }}}\leq \biggl(t_{q} + \frac{s_{q}-a_{qp}r_{p}}{r_{q}} \biggr)x_{q} + \frac{a_{qp}r_{p}}{r_{q}}x_{p}. $$

By (4), (5), and $\rho(B) - t_{p} > 0$, $\rho(B) - t_{q} > 0$, we have

$$ \bigl(\rho(B)-t_{p}\bigr) \biggl(\rho(B)- t_{q} - \frac{s_{q}-a_{qp}r_{p}}{r_{q}} \biggr)\leq\frac{s_{p}a_{qp}}{r_{q}}. $$

Therefore,

6 $$ \rho(B)\leq\frac{t_{p}+t_{q} + \frac{s_{qp}}{r_{q}}+\sqrt{ (t_{p}-t_{q} - \frac{s_{qp}}{r_{q}} )^{2}+\frac{4s_{p}a_{qp}}{r_{q}}}}{2}. $$

This must be true for every $p\neq q$. Then

7 $$ \rho(B)\leq\mathop{\min} _{j \ne q} \frac{t_{j}+t_{q} + \frac {s_{qj}}{r_{q}}+\sqrt{ (t_{j}-t_{q} - \frac{s_{qj}}{r_{q}} )^{2}+\frac {4s_{j}a_{qj}}{r_{q}}}}{2}. $$

This must be true for any $q\in N$. Then

8 $$ \rho(B)\leq\mathop{\max} _{1 \leq i \leq n} \mathop{\min} _{j \ne i} \biggl\{ \frac{t_{i}+t_{j}+\frac{s_{ij}}{r_{i}}+\sqrt{ (t_{i}-t_{j}+\frac{s_{ij}}{r_{i}} )^{2}+\frac{4s_{j}a_{ij}}{r_{i}}}}{2}, a_{ij}\neq 0 \biggr\} . $$

Lower bounds: Let $x_{p}>0$ be an arbitrary component of *x*, $x_{q}=\min\{ x_{k}, 1\leq k \leq n\}$. Obviously, $p\neq q$, $a_{pq}\neq0$. By $R^{-1}BRx = \rho(B)x$, we have

9 $$ \rho(B)x_{p}=t_{p}x_{p}+ \sum _{k = 1,k \ne p}^{n} {\frac{{a_{pk} r_{k} x_{k} }}{{r_{p} }}}\geq t_{p}x_{p} + \frac{x_{q}}{r_{p}}\sum _{k = 1}^{n} a_{pk} r_{k}\geq t_{p}x_{p} + \frac{x_{q}s_{p}}{r_{p}}. $$

Similarly, we have

10 $$ \rho(B)x_{q}=t_{q}x_{q}+ \sum _{k = 1,k \ne q}^{n} {\frac{{a_{qk} r_{k} x_{k} }}{{r_{q} }}}\geq \biggl(t_{q} + \frac{s_{q}-a_{qp}r_{p}}{r_{q}} \biggr)x_{q} + \frac{a_{qp}r_{p}}{r_{q}}x_{p}. $$

By (9), (10), and $\rho(B) - t_{p} > 0$, $\rho(B) - t_{q} > 0$, we have

11 $$ \bigl(\rho(B)-t_{p}\bigr) \biggl(\rho(B)- t_{q} - \frac{s_{q}-a_{qp}r_{p}}{r_{q}} \biggr)\geq\frac{s_{p}a_{qp}}{r_{q}}. $$

Therefore,

12 $$ \rho(B)\geq\frac{t_{p}+t_{q} + \frac{s_{qp}}{r_{q}}+\sqrt{ (t_{p}-t_{q}-\frac{s_{qp}}{r_{q}} )^{2}+\frac{4s_{p}a_{qp}}{r_{q}}}}{2}. $$

This must be true for every $p\neq q$. Then

13 $$ \rho(B)\geq\mathop{\max} _{j \ne q} \frac{t_{j}+t_{q} + \frac {s_{qj}}{r_{q}}+\sqrt{ (t_{j}-t_{q}-\frac{s_{qj}}{r_{q}} )^{2}+\frac {4s_{j}a_{qj}}{r_{q}}}}{2}. $$

This must be true for all $q\in N$. Then

14 $$ \rho(B)\geq\mathop{\min} _{1 \leq i \leq n} \mathop{\max} _{j \ne i} \biggl\{ \frac{t_{i}+t_{j}+\frac{s_{ij}}{r_{i}}+\sqrt{ (t_{i}-t_{j}+\frac{s_{ij}}{r_{i}} )^{2}+\frac{4s_{j}a_{ij}}{r_{i}}}}{2}, a_{ij}\neq 0 \biggr\} . $$

From (4), (5), and $x_{p}>0$ as an arbitrary component of *x*, we get $x_{k}=x_{q}=x_{p}$ for all *k*. Then we see easily that the right equality holds in (8) for $t_{i}+\frac{s_{i}}{r_{i}}= t_{j}+\frac{s_{j}}{r_{j}}$ for any distinct $i,j \in N$. The proof of the left equality in (3) is similar to the proof of the right equality, and we omit it here.

Thus, we complete the proof. □

## Signless Laplacian spectral radius of a graph

In this section, we will apply Theorem 2.1 to obtain some new results on the signless Laplacian spectral radius $\rho(G)$ of a graph.

### Theorem 3.1

*Let* $G = (V, E)$ *be a simple connected graph on* *n* *vertices*. *Then*

15 $$\begin{aligned} & \mathop{\min} _{1 \le i \le n} \mathop{\max} _{ i \sim j } \biggl\{ \frac{d_{i}+ 2d_{j} -1+ \sqrt {(d_{i}-2d_{j}+1)^{2}+ 4d_{i}}}{2} \biggr\} \\ &\quad\leq\rho(G) \leq\mathop{\max} _{1 \le i \le n} \mathop{\min} _{ i \sim j } \biggl\{ \frac{d_{i}+ 2d_{j} -1+ \sqrt{(d_{i}-2d_{j}+1)^{2}+ 4d_{i}}}{2} \biggr\} . \end{aligned}$$

*Moreover*, *one of the equalities in* (15) *holds if and only if* *G* *is a regular graph*.

### Proof

We apply Theorem 2.1 to $Q(G)$. Let $t_{i}=0$ for any $i \in N$. Then $f(i,j)= \frac{d_{i}+ 2d_{j} -1+ \sqrt {(d_{i}-2d_{j}+1)^{2}+ 4d_{i}}}{2}$. Thus (15) holds.

And the equality holds in (15) for regular graphs if and only if *G* is a regular graph. □

### Remark 3.1

Obviously, we have

$$\begin{aligned} &\mathop{\max} _{1 \le i \le n} \mathop{\min} _{ i \sim j } \biggl\{ \frac{d_{i}+ 2d_{j} -1+ \sqrt{(d_{i}-2d_{j}+1)^{2}+ 4d_{i}}}{2} \biggr\} \\ &\quad \leq\mathop{\max} _{ i \sim j} \biggl\{ \frac{d_{i}+ 2d_{j} -1+ \sqrt{(d_{i}-2d_{j}+1)^{2}+ 4d_{i}}}{2} \biggr\} . \end{aligned}$$

That is to say, our upper bound in Theorem 3.1 is always better than the upper bound (1) in [4].

### Theorem 3.2

*Let* $G = (V, E)$ *be a simple connected graph on* *n* *vertices*. *Then*

16 $$ \rho(G) \geq\mathop{\min} _{1 \le i \le n} \mathop{\max} _{ i \sim j } \biggl\{ {\frac{{d_{i} + d_{j} + m_{j} - {{d_{i} }/ {d_{j} + \sqrt{ ( {d_{i} - d_{j} - m_{j} + {{d_{i} } / {d_{j} }}} ) + 4d_{i} } }}}}{2}} \biggr\} $$

*and*

17 $$ \rho(G) \leq\mathop{\max} _{1 \le i \le n} \mathop{\min} _{ i \sim j } \biggl\{ {\frac{{d_{i} + d_{j} + m_{j} - {{d_{i} }/ {d_{j} + \sqrt{ ( {d_{i} - d_{j} - m_{j} + {{d_{i} }/ {d_{j} }}} ) + 4d_{i} } }}}}{2}} \biggr\} . $$

*Moreover*, *one of the equalities in* (16), (17) *holds if and only if* *G* *is a regular graph or a bipartite semi*-*regular graph*.

### Proof

We apply Theorem 2.1 to $Q(G)$. Let $t_{i}=d_{i}$, $s_{i} =\sum_{j = 1}^{n} {a_{ij} r_{j} } = d_{i}m_{i}$ for any $1 \leq i \leq n$. Then $f(i,j)= {\frac{{d_{i} + d_{j} + m_{j} - {{d_{i} }/ {d_{j} + \sqrt{ ( {d_{i} - d_{j} - m_{j} + {{d_{i} }/ {d_{j} }}} ) + 4d_{i} } }}}}{2}}$. Thus (16), (17) hold.

And the equality holds if and only if *G* is a regular graph or a bipartite semi-regular graph. □

## Signless Laplacian spectral radius of a digraph

In this section, we will apply Theorem 2.1 to obtain some new results on the signless Laplacian spectral radius $\rho(\overrightarrow{G})$ of a digraph.

### Theorem 4.1

*Let* $\overrightarrow{G} = (V, E)$ *be a strong connected digraph on* *n* *vertices*. *Then*

18 $$\begin{aligned} &\mathop{\min} _{1 \le i \le n} \mathop{\max} _{ i \sim j} \biggl\{ \frac{d_{i}^{+}+ 2d_{j}^{+} -1+ \sqrt {(d_{i}^{+}-2d_{j}^{+}+1)^{2}+ 4d_{i}^{+}}}{2} \biggr\} \\ &\quad \leq\rho(\overrightarrow{G}) \leq\mathop{\max} _{1 \le i \le n} \mathop{\min} _{ i \sim j} \biggl\{ \frac{d_{i}^{+}+ 2d_{j}^{+} -1+ \sqrt{(d_{i}^{+}-2d_{j}^{+}+1)^{2}+ 4d_{i}^{+}}}{2} \biggr\} . \end{aligned}$$

*Moreover*, *one of the equalities in* (18) *holds if and only if* $\overrightarrow{G}$ *is a regular digraph*.

### Proof

We apply Theorem 2.1 to $Q(\overrightarrow{G})$. Let $t_{i}=0$ for any $1 \leq i \leq n$. Then $f(i,j)=\frac{d_{i}^{+}+ 2d_{j}^{+} -1+ \sqrt{(d_{i}^{+}-2d_{j}^{+}+1)^{2}+ 4d_{i}^{+}}}{2}$. Then the inequality (18) holds.

And the equality holds in (18) if and only if $\overrightarrow{G}$ is a regular digraph. □

### Remark 4.1

Obviously, we have

$$\begin{aligned} &\mathop{\max} _{1 \le i \le n} \mathop{\min} _{ i \sim j} \biggl\{ \frac{d_{i}^{+}+ 2d_{j}^{+} -1+ \sqrt{(d_{i}^{+}-2d_{j}^{+}+1)^{2}+ 4d_{i}^{+}}}{2} \biggr\} \\ &\quad \leq\mathop{\max} _{ i \sim j} \biggl\{ \frac{d_{i}^{+}+ 2d_{j}^{+} -1+ \sqrt{(d_{i}^{+}-2d_{j}^{+}+1)^{2}+ 4d_{i}^{+}}}{2} \biggr\} . \end{aligned}$$

That is to say, our upper bound in Theorem 4.1 is always better than the upper bound (2) in [5].

### Theorem 4.2

*Let* $\overrightarrow{G} = (V, E)$ *be a strong connected digraph on* *n* *vertices*. *Then*

19 $$ \rho(\overrightarrow{G}) \geq\mathop{\min} _{1 \le i \le n} \mathop{\max} _{ i \sim j} \biggl\{ {\frac{{d_{i}^{+} + d_{j}^{+} + m_{j}^{+} - {{d_{i}^{+} }/ {d_{j}^{+} + \sqrt{ ( {d_{i}^{+} - d_{j}^{+} - m_{j}^{+} + {{d_{i}^{+} }/ {d_{j}^{+} }}} ) + 4d_{i}^{+} } }}}}{2}} \biggr\} $$

*and*

20 $$ \rho(\overrightarrow{G}) \leq\mathop{\max} _{1 \le i \le n} \mathop {\min} _{ i \sim j} \biggl\{ {\frac{{d_{i}^{+} + d_{j}^{+} + m_{j}^{+} - {{d_{i}^{+} }/ {d_{j}^{+} + \sqrt{ ( {d_{i}^{+} - d_{j}^{+} - m_{j}^{+} + {{d_{i}^{+} } / {d_{j}^{+} }}} ) + 4d_{i}^{+} } }}}}{2}} \biggr\} . $$

*Moreover*, *one of the equalities in* (19), (20) *holds if and only if* $\overrightarrow{G}$ *is a regular digraph or a bipartite semi*-*regular digraph*.

### Proof

We apply Theorem 2.1 to $Q(\overrightarrow{G})$. Let $t_{i}=d_{i}^{+}$, $s_{i} =\sum_{j = 1}^{n} {a_{ij} r_{j} } = d_{i}^{+}m_{i}^{+}$ for any $1 \leq i \leq n$. Then $f(i,j)={\frac{{d_{i}^{+} + d_{j}^{+} + m_{j}^{+} - {{d_{i}^{+} }/ {d_{j}^{+} + \sqrt{ ( {d_{i}^{+} - d_{j}^{+} - m_{j}^{+} + {{d_{i}^{+} }/ {d_{j}^{+} }}} ) + 4d_{i}^{+} } }}}}{2}}$. Thus (19), (20) hold.

One sees easily that the equality holds if and only if $\overrightarrow {G}$ is a regular digraph or a bipartite semi-regular digraph. □

## Conclusion

In this paper, we give some new sharp upper and lower bounds for the spectral radius of a nonnegative irreducible matrix. Using these bounds, we obtain some new and improved bounds for the signless Laplacian spectral radius of a graph or a digraph which are better than the bounds in [4, 5].
